# Overexpression of Myocilin in the *Drosophila* Eye Activates the Unfolded Protein Response: Implications for Glaucoma

**DOI:** 10.1371/journal.pone.0004216

**Published:** 2009-01-16

**Authors:** Mary Anna Carbone, Julien F. Ayroles, Akihiko Yamamoto, Tatiana V. Morozova, Steven A. West, Michael M. Magwire, Trudy F. C. Mackay, Robert R. H. Anholt

**Affiliations:** 1 Department of Genetics, North Carolina State University, Raleigh, North Carolina, United States of America; 2 Department of Zoology, North Carolina State University, Raleigh, North Carolina, United States of America; 3 W. M. Keck Center for Behavioral Biology, North Carolina State University, Raleigh, North Carolina, United States of America; Indiana University, United States of America

## Abstract

**Background:**

Glaucoma is the world's second leading cause of bilateral blindness with progressive loss of vision due to retinal ganglion cell death. Myocilin has been associated with congenital glaucoma and 2–4% of primary open angle glaucoma (POAG) cases, but the pathogenic mechanisms remain largely unknown. Among several hypotheses, activation of the unfolded protein response (UPR) has emerged as a possible disease mechanism.

**Methodology / Principal Findings:**

We used a transgenic *Drosophila* model to analyze whole-genome transcriptional profiles in flies that express human wild-type or mutant MYOC in their eyes. The transgenic flies display ocular fluid discharge, reflecting ocular hypertension, and a progressive decline in their behavioral responses to light. Transcriptional analysis shows that genes associated with the UPR, ubiquitination, and proteolysis, as well as metabolism of reactive oxygen species and photoreceptor activity undergo altered transcriptional regulation. Following up on the results from these transcriptional analyses, we used immunoblots to demonstrate the formation of MYOC aggregates and showed that the formation of such aggregates leads to induction of the UPR, as evident from activation of the fluorescent UPR marker, *xbp1-EGFP*.

**Conclusions / Significance:**

Our results show that aggregation of MYOC in the endoplasmic reticulum activates the UPR, an evolutionarily conserved stress pathway that culminates in apoptosis. We infer from the *Drosophila* model that MYOC-associated ocular hypertension in the human eye may result from aggregation of MYOC and induction of the UPR in trabecular meshwork cells. This process could occur at a late age with wild-type MYOC, but might be accelerated by MYOC mutants to account for juvenile onset glaucoma.

## Introduction

Primary open angle glaucoma (POAG, OMIM #137760) is the most common form of the heterogeneous group of optic neuropathies known as glaucoma [1]. POAG is generally characterized by impeded outflow of aqueous humor from the anterior eye chamber, resulting in elevated intraocular pressure (IOP) and death of retinal ganglion cells [2], [3].

The human glaucoma-associated myocilin (MYOC) protein has been associated with congenital glaucoma and with a small percentage of POAG cases [4], [5]. MYOC is a 57 kDa secreted glycoprotein of 504 amino acids encompassing two major domains: a coil-coiled myosin-like domain near the N-terminus and an olfactomedin-like domain (amino acids 245–504) near the C-terminus [6], [7]. Its function is unknown and the mechanisms by which mutations in MYOC cause glaucoma are not understood.

Several lines of evidence indicate that MYOC-associated glaucoma may be attributed to a gain-of-function disease model such that intracellular protein accumulation leads to cellular toxicity and cell death [8]–[13]. Mutant forms of MYOC that are introduced in cultured human trabecular meshwork cells and are not secreted accumulate as aggregates in the ER (ER) [12]. This results in the upregulation of 78 kDa glucose-regulated protein (GRP78) and protein disulfide isomerase (PDI), two proteins of the UPR pathway [14]. Another study investigated secretion of 35 MYOC variants in transfected COS-7 and immortalized human trabecular meshwork cell lines [9]. In either cell line, 20/35 of the MYOCs were not secreted into the cell culture medium. All 20 of these are disease-causing polypeptides in POAG and all have mutations in the olfactomedin domain of MYOC implying that the integrity of this domain is necessary for proper folding and that interference of MYOC export due to mutations in the olfactomedin domain may result in intracellular accumulation of misfolded proteins [9]. The solubility of MYOC complexes was also investigated by stably co-expressing GFP-tagged wild-type or mutant (C245Y or P370L) MYOC with transiently expressed FLAG-tagged wild-type MYOC in CHO-K1 and HEK293 cells. Complexes of GFP-C245Y or P370L and FLAG-tagged wild-type MYOC that were not secreted in the culture medium formed Triton X-100 insoluble complexes, which were retained in the rough ER and aggregated to form inclusion bodies. These MYOC aggregates induced the UPR proteins GRP78, PERK and CHOP/GADD153 which results in the activation of caspases 12 and 3, and eventually elicits the apoptosis pathway [15]. These studies suggest that accumulation of misfolded MYOC in the ER can trigger the UPR cascade thereby compromising trabecular meshwork cell function which would result in IOP and glaucoma.

*Drosophila melanogaster* has provided powerful genetic models for several neurodegenerative diseases, including Alzheimer's disease, Parkinson's disease, Huntington's disease [17], and retinal degeneration [16], [17]. Previously, a comparative genomic approach was developed in *Drosophila* by overexpressing myocilin in transgenic flies under an eye specific *gmr* promoter. Here, we have used whole-genome transcriptional profiling in this model to assess whether overexpression of wild-type and mutant forms of MYOC in the *Drosophila* eye results in activation of the UPR. Indeed, components of the UPR feature prominently among transcripts with altered expression in the transgenic flies. To verify that activation of the UPR is indeed correlated with the morphological and behavioral impairments observed in the transgenic flies, we demonstrate directly the formation of MYOC aggregates and visualize induction of the UPR in the larval eye imaginal discs with the ER-stress response marker, x box protein 1 (xbp1)-EGFP, previously used to detect activation of the UPR in the *Drosophila* model of retinal degeneration [16], [17].

Evolutionary conservation of the UPR together with previous reports that implicate this pathway in the etiology of glaucoma in human trabecular meshwork cells [9], [12], [14] support the notion that comparative genomics and proteomics studies that utilize the *Drosophila* model may provide new insights into conserved cellular pathways that are associated with human ocular hypertension and glaucoma, such as identification of genetic modifiers of MYOC-induced cellular stress.

## Results

### Generation of transgenic flies expressing wild-type and mutant MYOC

**Figure 1:**
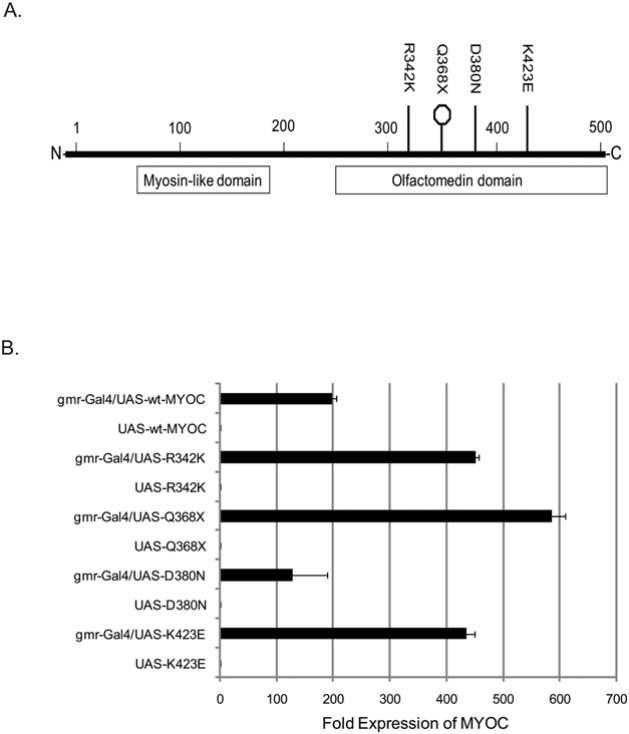
Expression of wt-MYOC and mutant MYOCs in transgenic flies. A) Diagram of the MYOC polypeptide showing the position of the amino acid substitutions (R342K, Q368X, D380N and K423E) that were created by site-directed mutagenesis. The Q368X mutant is predicted to result in a premature stop codon (octagon). Also shown are the N-terminal myosin-like domain and the C-terminal olfactomedin domain. B) Gene expression by quantitative PCR of human wt- MYOC and mutant MYOCs in heads of transgenic flies. Expression of MYOC is evident where the Gal4 driver is combined with the UAS-transgenes, but not in the parental lines.

We generated transgenic flies expressing wt-MYOC and mutant MYOC in their eyes and confirmed expression of the transgenes by quantitative RT-PCR (Figure 1). These analyses revealed greater than 50-fold expression of MYOC in F1 flies compared to their specific UAS-MYOC parental control (Figure 1B). The *gmr-Gal4/UAS-Q368X* and *gmr-Gal4/UAS-D380N* hybrids had the greatest level of expression (more than 150-fold) compared to the parental strain. Differences in expression levels between the five MYOC transgenic fly strains could be attributed to positional effects.

### Eye phenotypes and behavioral defects in transgenic flies

**Figure 2:**
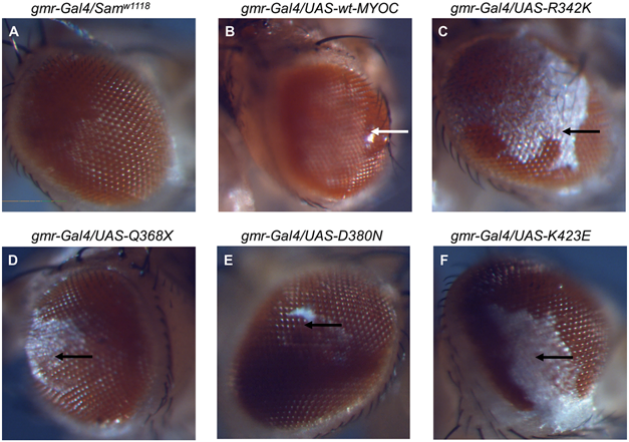
Eye phenotypes and phototactic responses of wild-type and mutant MYOC transgenic flies. Fluid discharge from eyes of transgenic flies. The normal appearance of eyes from a fly of the *gmr-Gal4/Sam* ^w1118^ control line is shown in panel A. A fly eye expressing the wt-MYOC with liquid discharge (white arrow) is shown in panel B. The flies expressing mutant MYOCs displaying a white salt residue (black arrows) after drying of the liquid residue (panels C–F).

Transgenic flies expressing mutant forms of MYOC developed the same fluid discharge phenotype reported previously for flies expressing wt-MYOC [18]. This intermittent fluid discharge through the lenses of the compound eye could be considered a proxy for increased intraocular pressure, which cannot be measured directly in the *Drosophila* eye (Figure 2). It has previously been shown that expression of GAL4 under the control of *gmr* has an effect on *Drosophila* eye development. Homozygotes have a disorganized ommatidial array and the levels of apoptosis in third-instar larval eye imaginal discs are significantly higher compared to wild-type discs [19]. Consistent with these results, our control line (*gmr-Gal4/Sam^w1118^*) displays some fluid-discharge, but this is much less prominent than seen in the MYOC-expressing flies.

**Figure 3:**
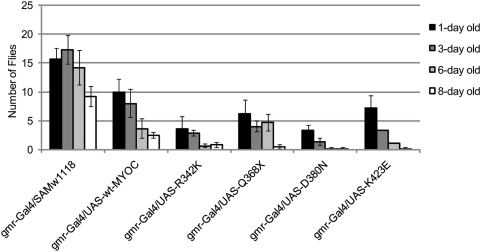
Phototactic behavior of MYOC-expressing transgenic flies. Progressive visual impairment of the MYOC-expressing flies compared to the *gmr-Gal4/Sam* ^w1118^ control line is observed.

We examined visual impairment of the MYOC transgenic flies by a behavioral assay for phototaxis using 1, 3, 6 and 8 day old flies (Figure 3 and Table S4). As expected, *Samarkand w^1118^* flies were blind and unresponsive to light (data not shown). The *gmr-Gal4/Samw^1118^* control strain contains a single copy of the *miniwhite* gene, and an average of 28% (18%) of the total population of 6-day old (8-day old) control flies moved towards the light source by the end of the 1 min trials. The *gmr-Gal4/UAS-MYOC* transgenic flies contain two functional *miniwhite* alleles and would be expected to have better visual function than the driver alone. The wt-MYOC and MYOC mutants, however, showed a progressive decline in phototactic behavior and only 5% (wt-MYOC) to 0.3% (D380N and K423E) of 8-day old flies moved towards the light source (Figure 3). To compare differences between classes (genotype, age, and genotype x age), we calculated pairwise contrasts and showed that mutant and wild-type MYOC expressing flies were significantly impaired in their phototactic behavior compared to the control at ages 3, 6 and 8-days with *P*-values ranging from *P*<0.00005 (for the D380N *vs* control at age = 3 days) to *P* = 0.018 (for the R342K *vs* control at age = 8 days).

### Transcription profiles in *Drosophila* eyes that express MYOC

MYOC-induced ocular secretion with progressive deterioration of visual function is reminiscent of the pathogenic sequelae that lead to the manifestation of MYOC-associated glaucoma. We hypothesized that conserved cellular pathways would be activated in the response to cellular stress in both the transgenic MYOC expressing flies and in compromised trabecular meshwork cells. To identify cellular mechanisms that are recruited to respond to overexpression of wild-type or mutant MYOC, we investigated the genome-wide expression profiles in response to transgenic MYOC expression and compared expression profiles of the parental UAS-MYOC lines and the MYOC-expressing hybrids.

**Figure 4:**
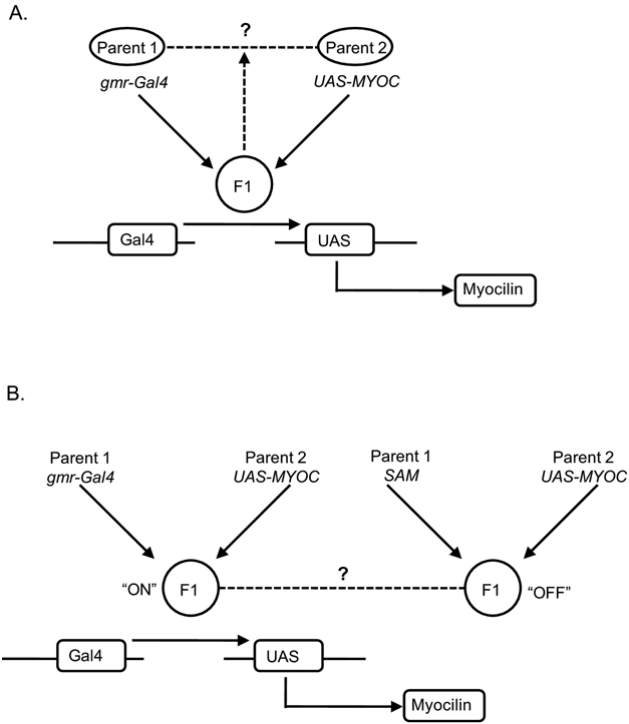
Diagrammatic representation of the expression microarray analysis. A) One-way ANOVA was used to compare differences between expression levels for each probe set in the F1 hybrid and the expected mean value of the two parents according to the model *Y = μ+G+e*, where *μ* is the overall mean, *G* is the effect of genotype, and *e* is the error variance. B) Two-way ANOVA was used to compare differences between expression levels for each probe set between F1 hybrids in which the UAS-transgene was ‘on’ or ‘off’ according to the model *Y = μ+E+C+E×C+e*, where *E* represents the effect of MYOC expression (‘on’ or ‘off’), *C* is the effect of the MYOC construct (wt-MYOC, R342K, Q368X, D380N, K423E), *E×C* is the effect due to the interaction between expression and each construct, and *e* is the error variance.

**Figure 5:**
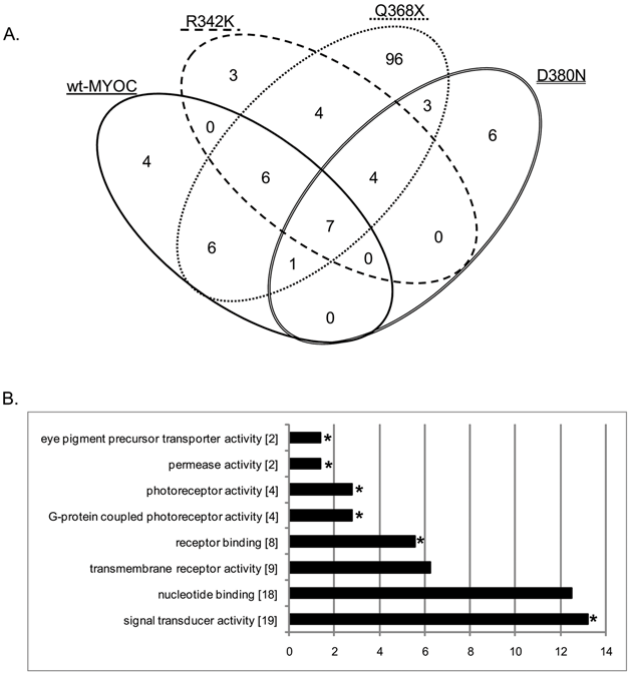
Probe sets with altered transcript abundance when expression in the F1 is compared to the predicted mean values of the parents. A) Venn diagram [64] depicting the overlap of probe sets among MYOC transgenes. Only one gene (1632731_at; *CG8613*) was differentially expressed in flies carrying the K423E construct and is not included in the diagram. The Q368X construct shows a uniquely high proportion of transcripts with altered expression (89%) that do not overlap with transcripts with altered expression in the other transgenic lines. B) Bar-chart showing the distribution of differentially expressed genes among Gene Ontology (GO) molecular function processes according to the analysis illustrated in Figure 4A. Parameters in DAVID [62] were set to GO level “all”, count threshold of 2 and EASE threshold of 0.1. The output is sorted by percentage. Asterisks refer to modified Fisher-Exact *P* values (*P*<0.05, *i.e.* strongly enriched in the annotation categories) and the numbers in brackets refer to the number of genes in the category.

First, we assessed probe sets that were differentially expressed in each *gmr-Gal4/UAS-MYOC* background, compared to the average expression of the homozygous *gmr-Gal4* and *UAS-MYOC* parental lines (Figure 4A). Summed across all five analyses we identified 197 probe sets (corresponding to 140 non-redundant genes) at an FDR<0.1 and in which the mean of the parental strains was significantly different (*P*<0.05) from their corresponding MYOC-expressing hybrid (Table 1 and Table S1). Ninety-seven of the 197 probe sets were upregulated (signal ratio: F1/[P1+P2]/2>1.0) and the remaining 100 were downregulated. Of the 140 genes, 76% were differentially regulated by one of the five different MYOC constructs (Figure 5A). The construct that showed the greatest disruption in genome-wide transcriptional regulation was the truncated mutant Q368X. The altered transcription profile in flies expressing the Q368X mutation showed the least overlap with those of flies expressing wt-MYOC or the R342K, D380N and K423E mutants (Figure 5A). Genes that showed altered expression when this construct was overexpressed; include *turtle*, *RpS24*, *Bruce*, *Osi6 and Obp57a*. To account for the possibility that gene disruption in any one construct might be due to positional effects of the insertion of the construct rather than the presence of the transgene, we focused on genes that were differentially regulated in three or more of the five transgenic constructs. Only 18 genes fulfilled this criterion. Five of these genes (*CG8613*, *Cyp6g1*, *Glut1*, *CG4210*, and *CG9772*) have human homologs (SPATA20, CYP3A7, SLC2A1, SAT2, and SKP2 respectively). Overrepresented molecular function categories include eye pigment precursor transporter activity, permease activity, photoreceptor activity and receptor binding (Figure 5B).

**Table 1:** Differential regulation of gene expression in eyes of transgenic flies that express wt-MYOC or mutant MYOCs, detected by one-way and two-way ANOVA*.

	Number of Genes with Altered Transcriptional Regulation			
	One-Way ANOVA	Two-Way ANOVA		
FDR	F1 vs Parental mean	Expression	Construct	E×C
0.1	140 (non-redundant)	7054	8272	8355
0.01	8	4673	6156	6111
0.001	0	2643	3117	2301
0.0001	0	1065	162	90
0.00001	0	482	19	4

* A diagrammatic representation of the analyses is shown in Figure 4.

Our initial analysis identified probe sets that are differentially expressed in each MYOC background, but is limited in power since each analysis is based on only nine arrays. We performed a second analysis which simultaneously compares gene expression levels of all five MYOC transgenes as homozygotes and as F1 g*mr-Gal4/UAS-MYOC* heterozygotes. This analysis considers expression of MYOC to be ‘off’ in the homozygous MYOC genotypes and ‘on’ in the heterozygous genotypes (Figure 4B). Using this approach we detected a total of 8063 probe-sets (5511 non-redundant probe-sets) with significant variation in transcript abundance (FDR<0.001; Table 1 and Table S2). Of these genes, 2643 genes (33%) were significantly different by expression, in other words, whether the expression of MYOC when turned “off” (parental P2 lines) is significantly different from when it is turned “on” via the *gmr* promoter (F1 hybrids). Fold level changes (Table S3) were estimated by calculating the signal ratio of F1/P2. This resulted in 2,118 genes that were up-regulated (signal ratio, F1/P2>1.00) and 525 genes that were down-regulated (signal ratio, F1/P2<1.00). Of the 2,643 genes that were significantly different by expression, 44% have human homologs. These included the five genes with human orthologs identified in the previous analysis, described above (FDR<0.001).

**Figure 6:**
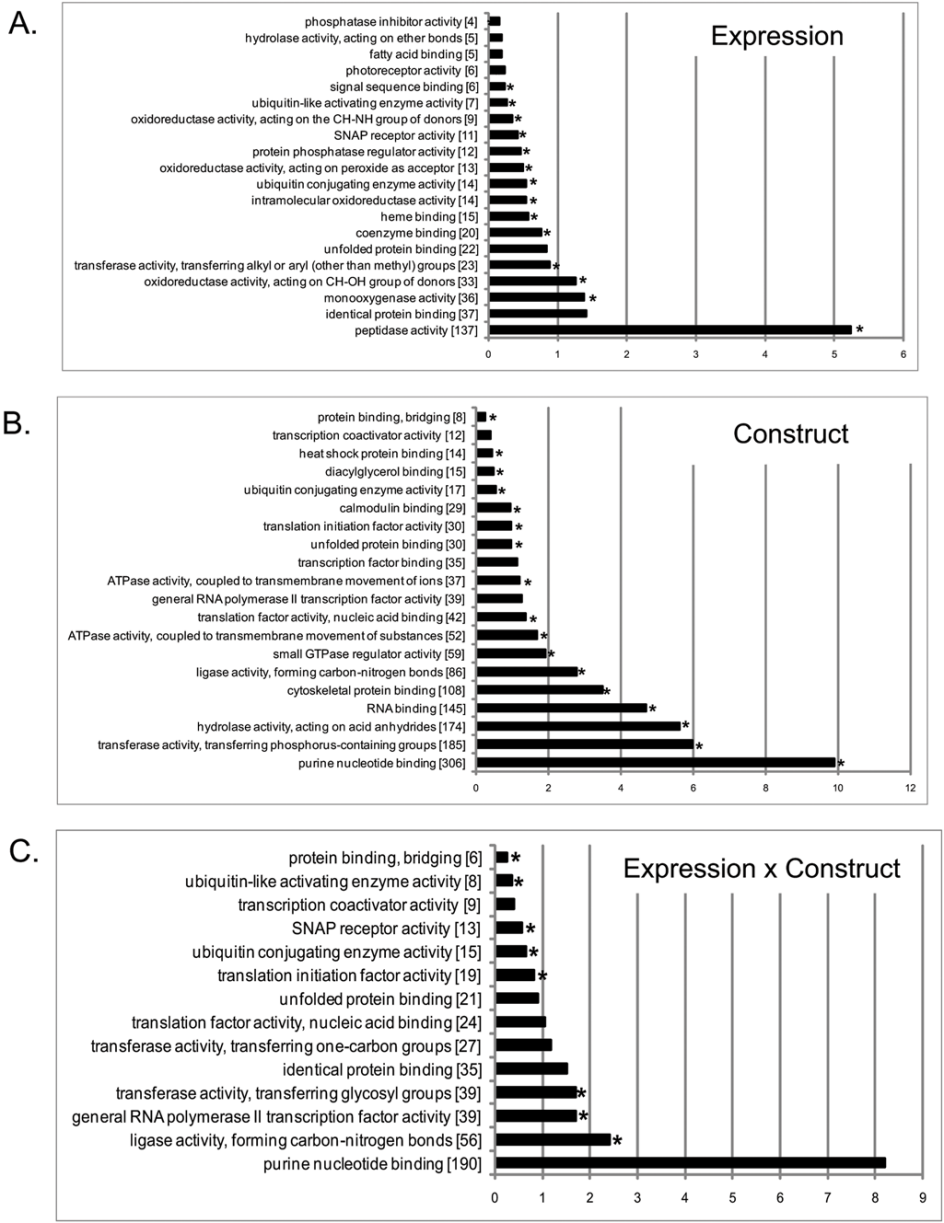
Gene ontology categories when transcript expression is analyzed across all genotypes comparing the ‘on’ and ‘off’ mode (Figure 4B). Bar-chart showing the distribution of differentially expressed genes among Gene Ontology (GO) molecular function processes, for each of the 3 terms in the two-way ANOVA (expression, A; construct, B; expression×construct, C). Parameters in DAVID [62] were set to GO level 3, count threshold of 2 and EASE threshold of 0.1. The output is sorted by percentage. Asterisks refer to modified Fisher-Exact *P-*values (*P*<0.05, *i.e.* strongly enriched in the annotation categories) and the numbers in brackets refer to the number of genes in the category.

We identified molecular function gene ontology categories that were enriched for each of the three terms of this two-way ANOVA (Figure 6). These categories include peptidase activity, photoreceptor activity, signal-sequence binding, oxidoreductase activities, transferase activity, heat shock protein binding, ubiquitin conjugating enzyme activity, unfolded protein binding, ATPase activities, SNAP receptor activity and ligase activity. Genes which encode peptidases include *Prosbeta2*, *Prosbeta3*, *Prosbeta5*, *Prosalpha7*, *Pros25*, *Pros26*, *Pros29 and Pros54*, all of which are proteasome components and were upregulated. Ubiquitin conjugating enzyme activity and unfolded protein binding were especially prominent molecular function GO categories, which were significantly enriched according to all three terms of the two-way ANOVA. *Drosophila* genes within the ubiquitin enzyme activity categories include *Bruce*, *UbcD2*, *UbcD4*, *UbcD6*, *UbcD10*, *Uba1*, and *Uev1A* with human orthologs BIRC6, UBE2E1, HIP2, UBE2A, UBE2L3, UBE1 and UBE2V1, respectively. The unfolded protein binding category includes the *Drosophila* genes *PEK*, *Hop*, *Hsc70-5*, *Gp93*, *CG7598* and *Crc* with human homologues EIF2AK3, STIP1, HSPA9B, HSP90B1, NDUFAF1, and CALR, respectively.

### Detection of MYOC aggregates

**Figure 7:**
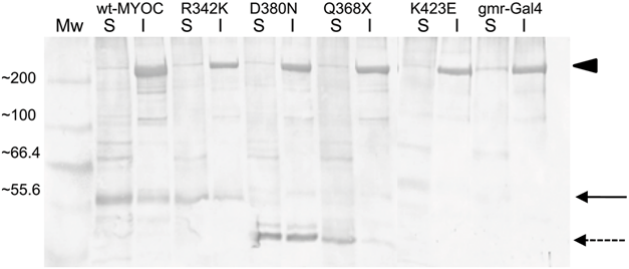
Western blot of soluble (S) and insoluble MYOC proteins (I) isolated from heads of transgenic flies. The wt-MYOC, R342K, Q368X and K423E represent the corresponding *gmr-Gal4/UAS-MYOC* genotypes; gmr-Gal4 designates the *gmr-Gal4/Sam* ^w1118^ control. The solid-arrow indicates the MYOC proteins that are expressed in the *gmr-GAL4/UAS-MYOC* heads at ∼57 kDa. The dashed-arrow indicates the Q368X-MYOC protein at ∼41 kDa. MYOC proteins recovered in the insoluble fraction represent aggregated proteins prior to treatment with SDS. The high molecular weight band that is also observed in the *gmr-Gal4/Sam* ^w1118^ control, likely represents cross-reactivity with the myosin heavy chain (arrow-head).

The induction of transcripts that encode components of the UPR and ubiquitination pathways suggested that aggregation of wt-MYOC and MYOC mutants might result in an ER stress response, which induces the UPR, as may occur in the human trabecular meshwork [12]. We used a MYOC antibody and Western blotting to identify the expressed proteins in homogenates from transgenic fly heads. Polyacrylamide gel electrophoresis and immunoblotting confirmed the presence of an immunoreactive protein of correct molecular weight of approximately 57 kDa (Figure 7) for wt-MYOC, as well as for the D380N, K423E, and R342K mutants. As expected, the Q368X MYOC mutant results in the translation of a truncated protein of about 41 kDa due to the presence of a premature stop codon at amino acid position 368 (Figure 7). To confirm the presence of MYOC aggregates in the transgenic flies, we fractionated homogenates from fly heads into soluble and insoluble fractions. The majority of wt-MYOC and the R342K, D380N and K423E MYOC mutants were recovered in the insoluble fraction, indicating extensive aggregation (Figure 7). We observed fewer aggregates of the Q368X protein, which lacks the C-terminal olfactomedin domain and was recovered mainly in the soluble fraction. In addition, we observed smaller molecular-weight soluble and insoluble immunoreactive polypeptides for the D380N construct, indicating that this protein may be unstable and undergoing degradation. No immunoreactive MYOC polypeptides were seen in the *gmr-GAL4* control. The *Drosophila* genome encodes a single olfactomedin-like protein (*CG6867*), which is expressed in the antenna and ocelli, but not in the adult compound eye (data not shown), and does not cross-react with our MYOC antibody. The high molecular weight band (>200 kDa) that is evident in the insoluble lanes likely corresponds to the *Drosophila* myosin heavy chain (CG31045, ∼240 kDa), which is homologous to the N-terminal (amino acids 74–176) of human MYOC [20].

### The UPR is activated in response to overexpression of MYOC

**Figure 8:**
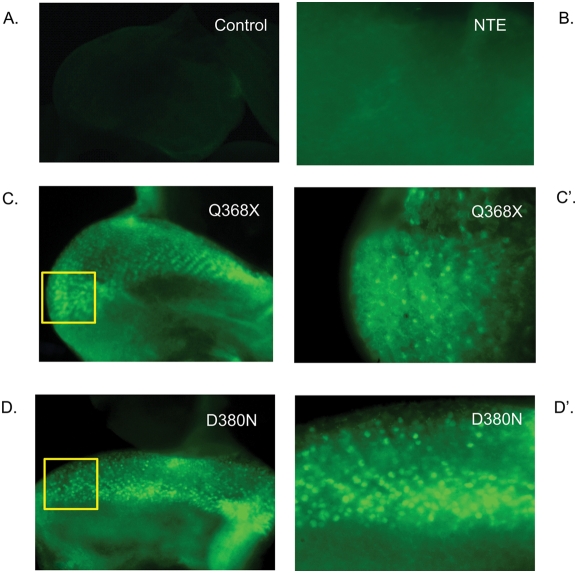
ER-stress activates *xbp1-EGFP* expression in MYOC-expressing transgenic flies. All panels show eye-imaginal discs from third-instar F1 larvae after crossing *gmr-Gal4*/*UAS-xbp1-EGFP* flies to the following lines: A) *gmr-Gal4/Sam* ^w1118^ (control), B) *gmr-Gal4*/*UAS-NTE*, C) *Gal4*/*UAS-Q368X*, D) *gmr-Gal4*/*UAS-D380N*. Panels C′ and D′ show 100× magnifications of the boxed areas in C and D, respectively. Punctate nuclear staining reflects activation of the UPR. Only background fluorescence is observed in flies that overexpress NTE (B) without the characteristic punctate nuclear staining.

To confirm activation of the UPR, we used a specific UPR marker, *xbp1-EGFP*, in which EGFP is expressed upon ER-stress in response to proteins that form aggregates in the ER [16]. We generated double-heterozygous transgenic flies that harbor the *xbp1-EGFP* marker and express the wt-MYOC and each of the four mutants (R342K, Q368X, D380N and K423E) using the *gmr-Gal4/UAS* system [21]. We also generated a line that expresses neuropathy target esterase (NTE) as a negative control. NTE is the human ortholog of *swiss cheese*, which is upregulated in flies that overexpress MYOC [18]. We did not observe activation of the UPR either in control flies or in NTE expressing flies, indicating that upregulation of NTE is a consequence rather than a cause of the MYOC-induced UPR (Figure 8). Activation of the *xbp1-EGFP* construct showed prominent punctate nuclear fluorescent staining in the larval eye-imaginal discs of flies expressing MYOC, especially those expressing the Q368X and D380N MYOC mutants (Figure 8), which show the highest expression levels in the transgenic flies (Figure 1B). Thus, MYOC aggregate formation appears to induce the UPR.

## Discussion

### *Drosophila* as a disease model

*Drosophila melanogaster* has emerged as a surprisingly powerful genetic model system for the study of quintessential human disorders such as Alzheimer's disease [22], Parkinson's disease [23], [24], Huntington's disease [25] and retinal degeneration [16]. Comparative genomic approaches using mutant or transgenic flies have been applied to investigate disease mechanisms under conditions in which both the genetic background and environmental conditions can be controlled.

MYOC is one of few genes that have been linked to the incidence of glaucoma [5], [26]–[30]. The Q368X mutation is predicted to result in a truncated MYOC-protein and was identified in two families during a screen of four different chromosome 1q-linked glaucoma families [30] and in 3/103 unrelated open angle glaucoma patients. A screen of 1703 POAG patients from five different populations representing three racial groups identified Q368X as the most common mutation, occurring in 1.6% of the probands, except among Japanese [31]. The D380N and R342K mutations were each detected in 2/90 West African POAG patients but not in 76 ethnically matched controls [32]. A French-Canadian family has been described in which both parents had glaucoma due to heterozygosity for the K423E mutation of the MYOC gene [33]. Like MYOC-associated glaucoma in humans (in particular juvenile onset), the fluid discharge phenotype observed in the transgenic flies that overexpress intact or mutant MYOC is likely to result from ocular hypertension and causes a rapid and progressive deterioration of visual function.

### Glaucoma and the UPR

Previous studies in cultured human trabecular meshwork cells have suggested that the UPR in response to aggregation of MYOC would represent one disease mechanism in the pathogenesis of intraocular pressure and glaucoma [13], [15].

The UPR signaling pathway is activated by ER stress leading to increased transcription of gene products which reduce the overall rate of protein translation while increasing the degradation of misfolded ER proteins through the ubiquitin-proteasome pathway. When these adaptive responses are not sufficient to relieve ER stress, apoptosis is induced [34]. Using transcription profiling in transgenic flies that express wt-MYOC and mutants of MYOC we indeed identified genes associated with the UPR, ubiquitination, apoptosis, and oxidative stress characteristic of pathways that can be triggered by ER stress as a consequence of misfolded protein accumulation [35]. The transcription profile data together with the detection of insoluble MYOC on Western blots suggests a mechanism by which misfolded MYOC accumulates in the ER, resulting in a cellular stress response that triggers a cascade of events which leads to the degradation of misfolded proteins via the ubiquitin-proteasome pathway. We visualized the ER-stress response directly with a UPR-specific marker, *xbp1-EGFP*, and observed expression of EGFP in the MYOC and mutant MYOC-expressing lines, but not in control flies (*gmr-Gal4/UAS-xbp1-EGFP*) or NTE-expressing flies. This indicates that the UPR pathway is indeed activated specifically by MYOC aggregates. Activation of the UPR was observed also in flies that express the Q368X mutant (Figure 8), even though less aggregation was observed in these transgenic flies (Figure 7). This is mostly likely due to the formation of weak aggregates which dissociate upon detergent solubilization and SDS-PAGE, since the truncated Q368X protein lacks the olfactomedin presumptive protein-protein interaction domain. It is also possible that accumulation of the Q368X protein leads to the UPR via a different mechanism, since it cannot be processed through the ER; previous studies have shown that truncated forms of MYOC are not secreted in the trabecular meshwork [8].

The misfolded protein aggregation hypothesis in the etiology of glaucoma is attractive as it places this disease in the same category of other neurodegenerative diseases that are associated with the formation of protein aggregates, including Huntington's disease, and Alzheimer's disease, which are also diseases that become manifest at an advanced age, like glaucoma. It should be noted that the normal function of MYOC may not be related to its pathogenic role in the trabecular meshwork. Neuronal olfactomedins have been implicated in early development of the nervous system [36], [37]. Thus, one can speculate that MYOC, a member of the olfactomedin family, may have a function primarily in early eye development, and that its expression in the adult eye is recapitulated only under conditions of stress.

Our studies show evidence that the wild-type and mutant MYOCs cause ocular fluid discharge, MYOC aggregation, and induction of the UPR and that these effects correlate with the magnitude of MYOC expression (Figures 1B, 7 and 8). This is consistent with previous evidence from animal models, including studies in rats [38] and glaucomatous dogs [39], [40] in which an increased level of MYOC is associated with increased intraocular pressure and severity of the disease. In addition, our results are consistent with studies in cultured human trabecular meshwork cells, which have proposed a cellular stress response as a possible mechanism in the pathogenesis of glaucoma [12], [13], [15], [41]. Studies in mice, however, have been equivocal in this regard. When a mouse MYOC Y423H mutant (analogous to the human Y437H MYOC allele) was introduced into the endogenous mouse MYOC locus, the Y423H mutant MYOC protein was not secreted into the aqueous humor, but accumulated in cells of the iridocorneal angle [42]. These mice, however, showed no evidence of ER stress, ocular hypertension or glaucoma at 18 months of age. However, intraocular pressure and/or the UPR pathway might have been detected eventually if the mice had been allowed to age longer. Other transgenic mice in which an Y423H MYOC mutant transgene was introduced displayed symptoms of glaucoma including elevated intraocular pressure and loss of retinal ganglion cells [43]. In these mice mutant Y423H MYOC formed complexes with wt-MYOC which prevented its secretion. A third study also showed that mice expressing mutant human MYOC (including Q368X and Y437H) in their eyes developed elevated intraocular pressure [44]. This study proposed that the observed intraocular pressure was due to the exposure of a cryptic C-terminal peroxisomal targeting signal type 1 receptor (PTS1R) site on the misfolded mutant MYOC molecule. Indeed, the *Drosophila* homolog of PTS1R, *CG14815*, is differentially expressed in our transgenic *Drosophila* model.

### Alternative pathogenic mechanisms

Is the UPR the only pathogenic mechanism involved in the induction of ocular hypertension? Oxidative stress may contribute to the pathogenesis of several neurodegenerative diseases, including glaucoma (for reviews see [45], [46]). Oxidative stress occurs as a result of excessive production of reactive oxygen species which overwhelms the antioxidant capacity of the cell and leads to damage of nucleic acids, proteins and lipids. Oxidoreductase activities emerged as enriched molecular function categories in the analysis of our transcription profiles (Figure 6). Under normal conditions, reactive oxygen species are removed by superoxide dismutase, glutathione peroxidase and catalase. All three enzymes were up-regulated at FDR<0.001 (Table S3). In addition, we identified 31 cytochrome P450s (including *Cyp6g1*, *Cyp6a23*, *Cyp12b2*), 25 of which were up-regulated. Mutations in CYP1B1 have been identified in numerous studies of congenital glaucoma [47]–[53].

### Conclusions

Targeted expression of human glaucoma-associated MYOC in the *Drosophila* eye results in a fluid extrusion phenotype reminiscent of ocular hypertension accompanied by visual behavioral impairments. Whole-genome transcriptional analysis identifies changes in transcript abundance in conserved cellular stress pathways, including the unfolded protein ER stress response. Immunoblotting reveals MYOC aggregates and activation of the UPR is demonstrated directly with a fluorescent reporter transgene. Comparative genomics studies using the fly model may provide insights into conserved cellular pathways that are associated with the etiology of human ocular hypertension and glaucoma.

## Materials and Methods

### Cloning of MYOC mutants

Four MYOC mutants were generated by site-directed mutagenesis using the QuickChange mutagenesis kit (Stratagene, La Jolla, CA) according to the manufacturer's protocol. The template used was the pMC2 vector [8] which is the human wild-type MYOC (wt-MYOC) cDNA cloned into the pCR2.1 vector (Invitrogen, Carlsbad, CA). The following primer pairs were used to generate the MYOC variants: R342K (5′-ccagggcgctgagtccaAaactgtcataagatatgagc and 5′-gctcatatcttatgacagttTtggactcagcgccctgg); Q368X (5′-ggagctggctaccacggaTagttcccgtattcttggggtggc and 5′-gccaccccaagaatacgggaactAtccgtggtagccagctcc); D380N (5′-ggggtggctacacggacattAacttggctgtggatgaagcagg and 5′-cctgcttcatccacagccaagtTaatgtccgtgtagccacccc); and K423E (5′-gggagacaaacatccgtGagcagtcagtcgcc and 5′-ggcgactgactgctCacggatgtttgtctccc). The capital letter in each primer denotes the base-pair that was mutated in order to generate the MYOC variant encoding the amino-acid substitution.

Each MYOC construct was excised from the pCR2.1 vector and cloned into the *Kpn*I and *Not*I sites of the p*UAST* transformation vector. The sequences of the cloned inserts were confirmed and the MYOC constructs were designated *pUAST-wt-MYOC*, *pUAST-R342K*, *pUAST-Q368X*, *pUAST-D380N* and *pUAST-K423E*. *P*-element transformation was performed according to standard procedures [54] in the *Samarkand w^1118^* strain (*Sam* ^w1118^) by the Model Systems Genomics group (Duke University, Durham, NC), as previously described [18]. To determine in which chromosome the *P*-element had inserted, crosses of homozygous p*UAST*-*MYOC* males to virgin-female double-balancer stocks (*w*; *CyO/Sp*; *TM3*, *Sb/H*) were carried out [55]. The resulting F1 or F2 hybrids revealed that the wt-MYOC and Q368X-MYOC transgenes were inserted into chromosome *2* while the other constructs (R342K, D380N, and K423E) were inserted into chromosome *3*.

Male flies carrying the *gmr-Gal4* driver (*P{GAL4-ninaE.GMR}12*, FlyBase ID FBti0002994) were obtained from the *Drosophila* stock center (Bloomington, IN). We assessed phenotypic effects of 11 genotypes: *gmr-Gal4*, *UAS-wtMYOC*, U*AS-R342K*, *UAS- D380N*, *UAS-K423E*, *UAS-Q368X*, and the five *gmr-Gal4/UAS-MYOC* heterozygous F1 hybrids in which MYOC is expressed in the eyes [18]. The F1 hybrids were generated by crossing *gmr-Gal4* males (P1) to *Sam^w1118^/pUAST-MYOC* virgin females (P2). In addition we generated flies that overexpress NTE under a UAS promoter. Expression was verified by RT-PCR. All flies were maintained at 25°C and 70% humidity, on a 12-hour light-dark cycle.

### Real-time Quantitative PCR

Total RNA from the same batch used for transcription profiling was treated with Dnase1(Invitrogen) and converted to cDNA with the High Capacity cDNA Archive Kit and random primers (Applied Biosystems, Foster City, CA), using 125 ng total RNA per 100 µl reaction for each sample. Each pre-formulated TaqMan Gene Expression Assay (Applied Biosystems) consists of two sequence-specific PCR primers and a TaqMan assay-FAM labeled MGB (minor groove binder) probe. Each TaqMan assay was performed in quadruplicate for each cDNA sample. Each replicate assay used 11.25 ng total cDNA in a 20 µl final volume. Assays were run with 2× Universal Master Mix without uracil-N-glycosylase (Applied Biosystems) on Applied Biosystems 7900 Fast Real-Time PCR System using universal cycling conditions (10 min at 95°C; 15 s at 95°C and 1 minute at 60°C, 40 cycles). The pre-formulated TaqMan assay (Applied Biosystems) for human MYOC (assay ID: Hs00165345_ml) was used. The *Drosophila GAPDH* TaqMan gene expression assay was used as the endogenous control (assay ID: Dm01841185_m1).

Data were normalized by subtracting the average C_T_ (threshold cycle) of GAPDH from each replicate to give the ΔC_T_. The expression level of each sample was calculated as 2^(−ΔCT±SD)^, where SD is the standard deviation.

### Phototaxis Response

Visual impairments among transgenic fly genotypes were quantified using a phototaxis assay. Behavioral assays for phototaxis were performed in the dark using a protocol similar to ones described previously [56], [57]. An 18×150 mm Pyrex test tube was wrapped with black electrical tape and joined by a rubber ring to a clear 18×150 mm Pyrex test tube. A halogen lamp (12 V, 20 W) was used as the light source and was placed at a height of 12 inches perpendicular to the tubes. Flies were dark-adapted for 10 minutes and then gently tapped into the dark-side of the double-tube. The double-tube was placed horizontal at table level and the halogen light was turned on. Flies were given 1 minute to move from the dark tube towards the light, and the number of flies that migrated towards the light was recorded. Three trials each were performed on each of two replicates with mixed male-female populations of 50 flies, aged 1, 3, 6 and 8 days. Data were analyzed by ANOVA, using PROC MIXED implemented in SAS 9.13 statistical software (Cary, NC), according to the model, *Y* = *μ*+*G*+*A*+*G*×*A*+R(G×A)+T(R)+*e*, where *Y* is the observed value, *μ* designates the overall mean, *G* ,*A*, *R* and *T* denote genotype, age, replicate, and trial, respectively, and *e* is the residual experimental error. The R(G×A) and T(R) terms were used as random effects in the model.

### Transcriptional profiling

RNA was extracted from 3 independent biological replicates per genotype with Trizol reagent (Invitrogen, Carlsbad, CA) from heads of adult flies harvested 3–5 days post-eclosion, reared under controlled conditions of temperature (25°C), humidity (70%) and light cycle (12 hr/ 12 hr) and deprived of food for 20 minutes prior to RNA extraction. Transcriptional profiles for all 11 genotypes were generated simultaneously for a total of 33 GeneChips. Biotinylated cRNA probes were prepared by one-cycle target labeling, hybridized to high-density oligonucleotide microarrays (*Drosophila* 2.0 genome arrays; Affymetrix, Inc., Santa Clara, CA) and visualized with a streptavidin-phycoerythrin conjugate as described in the Affymetrix GeneChip Expression Analysis 2000 technical manual, using internal references for quantification.

### Microarray data preprocessing

Expression data were extracted from the .cel files using the *Affy* package in R-bioconductor [58]. All arrays were quantile normalized to remove non-biological variation between arrays using the Robust Multi-chip Averaging (RMA) algorithm [59]. We analyzed the log2 signal intensities of the perfect match probes (PM data). We used the Microarray Suite (MAS) 5.0 [60] present/absent calls as a noise filter (absent in more than three samples with signal intensities less than 4.906) to remove absent and low hybridization features. The correlation between replicates was high, ranging from 0.9971 to 0.913 with a median of 0.987. The raw microarray data are deposited in the ArrayExpress database (http://www.ebi.ac.uk/microarray-as/ae/) under accession number E-MEXP-1179.

### Statistical analysis: detection of differentially expressed genes

We performed two statistical analyses to identify differentially expressed genes in the MYOC backgrounds. First, we compared expression levels of the *gmr-Gal4* and *UAS-MYOC* homozygous parental genotypes with that of the *gmr-Gal4/UAS-MYOC* F1 hybrids separately for each MYOC genotype. We performed one-way ANOVA for each probe set according to the model *Y = μ+G+e*, where *μ* is the overall mean, *G* is the fixed effect of genotype, and *e* is the error variance. All genes for which the effect of genotype was significant at a false discovery rate (FDR; [61]) of <0.1 were further tested to determine whether mean expression of the F1 hybrid was significantly different from the average of the two parental strains (*P*<0.05;[18]). This analysis identifies probe sets that are differentially expressed in each MYOC background, but is limited in power since each analysis is based on only nine arrays.

The second analysis simultaneously compared gene expression levels of all five MYOC transgenes as homozygotes and as F1 g*mr-Gal4/UAS-MYOC* heterozygotes. Expression of MYOC is ‘off’ in the homozygous MYOC genotypes and ‘on’ in the heterozygous genotypes. We analyzed these data by two-way ANOVA for each probe set according to the model *Y = μ+E+C+E×C+e*, where *E* represents the effect of MYOC expression (‘on’ or ‘off’), *C* is the effect of the MYOC construct (wt-MYOC, R342K, Q368X, D380N, K423E), *E×C* is the effect due to the interaction between expression and each construct, and *e* is the error variance. All effects were fixed. We analyzed probe sets that were significant for the main and interaction effects at FDR<0.001. Statistical analyses were implemented using SAS 9.13 software (Cary, NC).

Data mining was performed using the web-accessible program DAVID [62] which can be accessed at http://david.abcc.ncifcrf.gov/, the NetAffx Analysis Center (http://www.affymetrix.com/analysis/index.affx) and FlyBase (http://www.flybase.org, release 4.3).

### Western Blotting

Analysis of MYOC protein aggregates formed by wt-MYOC and mutant MYOC in the transgenic flies were examined by Western blotting from a homogenate of 10 fly heads by extracting both soluble and insoluble proteins, as described previously [8]. Briefly, ten heads from five-day old flies were homogenized with 100 µl of ice-cold extraction buffer (10 mM Tris-HCl, pH 7.5, 5 mM EDTA, 1% NP-40, 0.5% deoxycholate, 150 mM NaCl) containing 1% protease inhibitor cocktail (Sigma-Aldrich) and lysed for 30 minutes at 4°C. The samples were centrifuged at 13,000 rpm for 15 minutes at 4°C. The supernatants were collected and the insoluble pellets solubilized with 50 µl of 10 mM Tris-HCl, 1% SDS for 10 minutes at room temperature and the volume adjusted to 100 µl with extraction buffer. Laemmli sample buffer (Bio-Rad Laboratories, Hercules, CA) containing 5% β-mercaptoethanol (Sigma-Aldrich, St. Louis, MO) was added to the cell extracts and the samples were boiled for 10 min. The samples were centrifuged at 13,000 rpm for 10 min at 4°C and equal volumes of the supernatants were separated by 7.5% SDS-PAGE, transferred to 0.45 micron nitrocellulose membranes, and probed with a 1000-fold dilution of anti-human MYOC antibody (R&D systems, Minneapolis, MN). Bound antibody was visualized with an 8000-fold dilution of anti-goat IgG-alkaline phosphatase (Sigma-Aldrich) secondary antibody and visualized using nitroblue tetrazolium chloride/ 5-bromo-4-chloro-3′-indoylphosphate p-toluidine salt as the chromogenic substrate (Sigma-Aldrich).

### Immunohistochemistry

To visualize the ER-stress response in the MYOC-expressing flies, we crossed *gmr-Gal4-UAS-MYOC* lines with the *gmr-Gal4-UAS-Xbp1-EGFP* line to assess activation of the xbp1-EGFP marker [16]. Xbp1 is a component of the UPR pathway and splicing of the *xbp1* mRNA at the Ire-1 splice site upon activation of this pathway enables the expression of EGFP [16]. We dissected brains from third instar larvae and immunostained them as described previously [63] using rabbit anti-GFP (Invitrogen Molecular Probes) as the primary antibody (1∶250 dilution) followed by FITC-conjugated Goat anti-Rabbit secondary antibody (1∶500 dilution) (Jackson ImmunoResearch Laboratories, Inc.). After the brains were stained, we dissected the eye-imaginal discs and viewed them using a Zeiss Axioplan fluorescent microscope with digital image collection.

## Supporting Information

Table S1 ANOVA of differences in transcript abundance in MYOC expressing F1 transgenic flies compared to average values of the GAL4 and UAS parental lines.
(15.11 MB XLS)

Table S2 ANOVA of differences in transcript abundance in MYOC expressing F1 transgenic flies in the presence or absence of the gmr-GAL4 driver.
(15.90 MB XLS)

Table S3 Relative expression levels of probe sets in MYOC expressing F1 transgenic flies in the presence versus absence of the gmr-GAL4 driver.
(0.68 MB XLS)

Table S4 ANOVA of phototactic responses of control and transgenic flies as a function of age.
(0.07 MB XLS)
